# Teaching This Class Drives Me Nuts! - Examining the Person and Context Specificity of Teacher Emotions

**DOI:** 10.1371/journal.pone.0129630

**Published:** 2015-06-08

**Authors:** Anne C. Frenzel, Betty Becker-Kurz, Reinhard Pekrun, Thomas Goetz

**Affiliations:** 1 Department of Psychology, University of Munich, Munich, Germany; 2 Department of Empirical Educational Research, Konstanz, Germany; 3 Thurgau University of Teacher Education, Kreuzlingen, Switzerland; University of L'Aquila, ITALY

## Abstract

Teachers’ emotions are critically important for the quality of classroom instruction, and they are key components of teachers’ psychological well-being. Past research has focused on individual differences between teachers, whereas within-teacher variation across contexts has rarely been considered. As such, the present research addresses the long-standing yet unresolved person-situation debate pertaining to the emotional experiences of teachers. In two diary studies (*N* = 135, 70% female, and *N* = 85, 28% female), we examined the role of person, academic subject, and group of students for teacher emotions; focusing on three of the most salient emotions found in teachers: enjoyment, anger, and anxiety. Findings from multi-level analysis confirmed the person specificity of enjoyment, anger, and, in particular, anxiety. In addition, underscoring the existence of within-teacher variability, findings supported that teachers’ emotions considerably varied depending on the subject and group of students taught, particularly so for enjoyment and anger. Implications of the person and context specificity of teacher emotions are discussed in relation to assessments and intervention programs aiming to improve teachers’ emotional lives in the classroom.

## Introduction

Teacher emotions have attracted increasing empirical research attention in recent years. They have been shown to be critically important for the quality and effectiveness of classroom instruction, and are highly relevant for teachers’ psychological well-being [1–6]. Given the relevance of teacher emotions for a broad range of teacher and student outcomes, it becomes necessary to learn more about the intricacies of teachers’ emotional lives. Past research has focused on individual differences between teachers, whereas within-teacher variation across contexts has rarely been taken into account [7, 8, 9]. In the present study, we sought to explore three questions: Are teachers’ emotions unique to the individual teacher (person specific)? Are teachers’ emotions dependent on the subject they are teaching (subject specific)? Are teachers’ emotions dependent on the group of students they are teaching (group specific)? We chose to focus on three emotions that have been shown to be of particular salience for teachers in the classroom—namely, enjoyment, anger, and anxiety [9].

Insight into the degree to which teacher emotions are person, subject, and group specific has far-reaching implications for assessment and intervention applications. If teacher emotions are shown to be predominantly shaped by the person, then generic approaches to their assessment could be used (e.g., “How do you generally feel when teaching?”), and intervention programs could focus on general emotion-regulation techniques. In contrast, if teacher emotions also proved to be shaped by the subject and/or student group, then assessment should be tailored to these levels of specificity (e.g., “How do you feel when teaching math/when teaching this group of students?”), and interventions should be designed to attend to the peculiarities of teaching certain subjects or particular groups of students. To date, established scales for the assessment of teacher emotions, and programs aimed to optimize teachers’ emotional experiences are scarce. Thus, a further aim of the present research is to provide empirically supported recommendations for the development of such assessment instruments and intervention programs.

### The Person and Context Specificity of Teacher Emotions

It is a long-standing and widely-acknowledged assumption in psychology that a person’s behavior and experience is a function of both the person and their situation [10]. Nevertheless, the person-situation debate concerning the consistency of behaviors and experiences across situations is still a heated one [11]. For affect and emotional experiences in particular, researchers have been intrigued by the question of how much they vary from person to person, as well as how they fluctuate within each individual from one moment to the next [12]. In an attempt to resolve the debate, it has been argued that researchers should shift to narrower units of analysis that are strongly tied to social cognitive processes. This would also facilitate the understanding of the dynamics underlying individual differences [13, 14]. For emotional experiences, this perspective aligns with appraisal theories of emotions which suggest that individuals’ subjective judgments of situations—resulting from their expectancies, attributions, and values—are of primary importance for emotion arousal [15–20]. As such, it can be assumed that emotions are determined both by the person as the beholder of the lenses through which a given situation is perceived *and* by the affordances and constraints of the situation.

One way of addressing the person versus context specificity of emotions is to assess people’s emotional experience repeatedly over time (e.g., “At the moment, I feel…”), and then to explore the proportions of variance relative to the total variance that are due to either the individual respondent, or due to within-person momentary fluctuations. If the proportion of variance due to the individual respondent is high, then an emotion can be considered person specific [21]. This approach also allows exploration into whether specific contexts shape emotional experiences within individuals. If significant proportions of the variance are due to a clearly defined context, then it would appear that these contextual factors play a role in determining how people feel. For the present research, context was defined as the academic content (i.e., the academic subject taught) and social context (i.e., the particular group of students taught).

Another way of addressing the question of context specificity is to record people’s reports of their emotional experience in a one-shot design, while formulating items explicitly with respect to various different contexts (i.e., “In context A, I feel…”, “In context B, I feel…”). Then, correlations between context-specific emotion scores can be assessed. If correlations between context-specific emotion scores are low, inferences from a person’s emotional experiences in one context to another context cannot be made reliably, and thus the considered contextual factors seem to play a role in determining how people feel.

#### Person specificity of teacher emotions

Generally, it is widely accepted that emotions can be understood as dispositional traits, or in other words, that people differ from one another in the frequency and intensity with which they experience certain emotions [22–24]. As such, we assumed that also teachers’ emotional experiences during teaching are, to a considerable degree, person specific. For example, we assume that the intensity and frequency of a teacher’s anxiety while teaching is shaped by their general disposition to experience anxiety, and that teachers thus differ systematically from one another in their experiences of anxiety while teaching.

For students, the person specificity of emotions was explored in a study by Ahmed, van der Werf, Minnaert, and Kuyper [25]. These authors assessed students’ emotional experiences over the course of two weeks using daily diary entries and found that students’ emotions were person specific to some degree, but that they also strongly fluctuated within students across days (within-student variance relative to total variance was 51%, 59%, and 70% for enjoyment, anger, and anxiety, respectively).

In contrast, evidence of the person specificity of teacher emotions during teaching is greatly lacking. Past research, however, particularly from the context of teaching effectiveness, has focused on individual differences between teachers [8], and seems to implicitly assume that teacher emotions and related variables (e.g., burnout, teacher self-efficacy) are specific to the individual teacher. In the present research, we sampled teachers from a wide range of contexts in order to explore in-depth the person specificity of teachers’ enjoyment, anger, and anxiety.

#### Subject specificity and group specificity of teachers’ emotions

We propose that teacher emotions should systematically vary depending on the academic subject and the group of students they teach. These assumptions are grounded in appraisal theories of emotions which suggest that individuals’ subjective judgments of situations—for example their expectancies, attributions, self-concepts and self-efficacy, subjective values, or goals—are of primary importance for general emotion arousal [15–20] and have been shown to also apply to teacher emotions [3, 9]. To the extent that these appraisals vary based on the academic subject or group of students taught, appraisal theory upholds that teachers’ emotions should similarly be organized in subject-specific and group-specific ways.

Self-efficacy appraisals may be especially important antecedents of teacher emotions [3, 9, 26]. Both the academic subject and the group of students taught are likely key contextual factors that influence a teacher’s self-efficacy, and thus also their emotional experiences.

Underscoring the subject specificity of teachers’ self-efficacy, it has been proposed, though untested, that in order to adequately assess teaching self-efficacy, it is necessary to differentiate between teaching the sciences and teaching other subjects [27]. Underscoring the group specificity of teaching self-efficacy, empirical evidence suggests that teaching self-efficacy varies within teachers as a function of the specific group of students taught (within-teacher variability due to different groups of students taught was 44% and 21% as reported by [28, 29] respectively).

Goetz and colleagues argued along the same lines for students [30–32]. They suggested that to the extent that achievement task appraisals are organized in subject-specific ways, students’ achievement emotions should also be organized in subject-specific ways. Indeed, they provided consistent empirical evidence that students’ emotional experiences vary from subject to subject. Specifically, Goetz et al. (2007) demonstrated that correlations between subject-specific emotion scales were strikingly low for students. For example, they reported that across domains, correlations were virtually zero (e.g., disattenuated correlations of .02 in grade 8, and of .06 in grade 11, between mathematics and German anxiety). Additionally, even between related school subjects, disattenuated correlations were no higher than .60 (e.g., .54/.48/.60 between mathematics and physics enjoyment/anger/anxiety in grade 11, and .25/.28/.21 between German and English enjoyment/anger/anxiety in grade 8).

Direct empirical evidence of the subject and group specificity of teacher emotions is lacking. There are, however, some findings suggesting that teachers’ affect and emotions may be group specific. For example, in several studies, class-average student motivation was found to correlate positively with teachers’ positive experiences during teaching [3, 7, 33]. Furthermore, Kunter and colleagues [7] found that teachers’ experiences of enjoyment and enthusiasm during teaching correlated positively with class-average student enjoyment and negatively with class-average student disruption, thus concluding that teachers’ enjoyment and enthusiasm were context specific. However, further direct conclusions about the relative degree to which teacher emotions are group specific cannot be inferred from this evidence.

To summarize, we posited that emotions vary from teacher to teacher and thus show a certain amount of person specificity. In addition, we posited that teachers’ emotions are subject specific—a notion grounded in appraisal theory and consistent with evidence of the subject specificity in student emotion research. Finally, we hypothesized that teacher emotions would also be specific to the group of students taught—a notion also based on appraisal theory and consistent with the scattered empirical findings on the group specificity of teacher self-efficacy. So far, existing research does seem to assume that teacher emotions and related variables are specific to the individual teacher, but direct evidence of the degree of person specificity of teacher emotions is lacking, and within-teacher differences in terms of variations across subjects and student groups have not yet been addressed. For students’ emotions and related constructs, subject specificity has been extensively studied [30–32, 34, 35, 36], but for teachers, this is a virtually unexplored area in educational research.

### Objectives and Design

In order to determine the degree of person and context specificity of teachers’ emotions during teaching, we used a diary approach. Over the course of two weeks, teachers filled out an emotion diary directly after teaching their class periods. They were asked to report the enjoyment, anger, and anxiety they had just experienced in the previous class period. In contrast to classical recall-based questionnaire approaches, we anticipated that the diary approach would yield valid estimates of teachers’ experiences because teachers reported on momentary emotions rather than on recollected past teaching experiences [37].

We tested our hypotheses using two samples of teachers: (a) classroom teachers who teach numerous academic subjects to only one group of students within one school year (Study 1) and (b) subject teachers who only teach a few typically related subjects to numerous groups of students within one school year (Study 2). In Germany, classroom teachers are typically found in primary schools (Grundschule) and lower-track secondary schools (Hauptschule), whereas subject teachers are typically found in middle- and upper-track secondary schools (Realschule and Gymnasium, respectively). Accordingly, Study 1 included German primary and lower-track secondary school teachers and examined the person specificity and subject specificity of teacher emotions. Study 2 included a sample of German middle- and upper-track secondary school teachers, which made it possible to additionally examine group specificity as source of teacher emotion variance.

## Study 1: Classroom Teachers’ Emotions

Study 1 investigated the person and subject specificity of classroom teachers’ enjoyment, anger, and anxiety. Specifically, in this study, we focused on classroom teachers who taught German, mathematics, and science.

### Method

#### Ethics statement

The research reported herein was conducted in accordance with the ethical standards expressed in the Declaration of Helsinki and has received a formal waiver of ethical approval by the ethics committee of the Department of Psychology, LMU Munich. Participation in both studies was voluntary, written informed consent was obtained from all participants, and no identifiers that could link individual participants to their results were obtained. Hence, all the analyses were conducted on anonymous data.

#### Sample, procedure and measures

Participants were *N* = 135 classroom teachers (70% female) including 63 primary school teachers (97% female) and 72 lower-track secondary school (Hauptschule) teachers (47% female). Teachers’ mean age was 44.34 years (*SD* = 11.46; *range* = 26–64 years), with an average of 17.89 years of teaching experience (*SD* = 11.77; *range* = 1–42 years). These ages and gender distributions are in line with the documented teacher populations in the state of Bavaria [38].

Paper-and-pencil diaries were used to assess teachers’ emotions during teaching over the course of two weeks (i.e., ten school days). Teachers were informed per written instructions how to use the diaries. Assessment materials were distributed and collected by external, trained testing personnel (pre-service teachers) shortly before the middle of the school year.

Teachers reported their enjoyment, anger, and anxiety with one item each (respectively, "I enjoyed this class period”, “I was angry during this class period”, and “I was tense and nervous during this class period”, answered on a 4-point Likert scale ranging from 1 = *strongly disagree* to 4 = *strongly agree*). Teachers were required to fill in the diaries directly after each German, mathematics, and science class period. If circumstances prevented them from filling in their diaries, they were instructed to skip the diary entry (in order to avoid memory biases that would occur if diaries were filled out with too large a time lag between the actual class period and the diary report). We used single items due to requirements to limit administration time for the diaries. Single-item scales have been shown to be valid measures of affective constructs [39]. In total, teachers reported about an average of 19.6, 19.8, and 19.8 class periods in their diaries for enjoyment, anger, and anxiety, respectively (numbers varying due to missing data).

#### Data structure and strategy of data analysis

The design of Study 1 allowed for an investigation of the person and subject specificity of teachers’ emotions. Given that our data had a two-level hierarchical structure (diary entries nested in teachers), we applied multi-level analysis to determine the person specificity of classroom teachers’ emotions. Notably, subject did not constitute a hierarchical level because subjects and teachers were fully cross-classified in our data structure: Diary entries were nested within teachers, on the one hand, and within subjects, on the other. As such, subjects and teachers did not have hierarchical correspondence to each other (for details on cross-classified vs. hierarchically structured data, see e.g. [40], Chapter 7). We specified for each emotion a so-called “null model” or “intercept only model” in the hierarchical linear approach [40, 41]. This model provides estimates of the sizes of the variance at each hierarchical level as well as significance tests for whether the variances at each level significantly differ from zero. Taking the emotion of enjoyment as an example, the hierarchical linear model is formally represented as follows: The current experience of enjoyment of teacher *j* in class period *i* (enjoyment_ij_), equates to the average experience of enjoyment across the total sample (γ_00_), plus the teacher specific deviation (u_0j_), plus the class period specific deviation (e_ij_). It is assumed that the teacher specific and class period specific deviations are each normally distributed with a mean of 0, and are independent from one another. As such, the variance of u_0_ (σ²_u0_) is an indicator for the inter-individual variance between teachers. If this value differs significantly from 0, this implies that the emotion systematically varies from teacher to teacher [40], Chapter 2. We used the software package Mplus (Version 7.11) to process these analyses.

Furthermore, we applied correlation analyses to determine subject specificity of classroom teachers’ emotions. To this end, we aggregated across all period entries for all German, mathematics, and science classes that each teacher had reported about, resulting in subject-specific emotion scores for each teacher. We then determined the correlations across these scores for any two subjects.

### Results

#### Preliminary analyses

The overall mean values across all diary entries for all teachers were *M =* 3.10 for enjoyment (*SD* = 0.37), *M =* 1.63 for anger (*SD* = 0.40), and *M =* 1.33 for anxiety (*SD* = 0.35). Using the rationale proposed by Zelenski and Larsen [42] to judge discrete emotion frequency, we assumed that an emotion was present if the teachers reported at least a value of two on the 4-point Likert scale. We thus explored the emotion frequency, for each teacher, by calculating the proportion of class periods in which each of the emotions was present. On average, the teachers reported experiencing enjoyment in 97%, anger in 44%, and anxiety in 25% of all their class periods.

#### Person and subject specificity of classroom teachers’ emotions

Table 1 presents the variances for enjoyment, anger, and anxiety at the teacher and class period levels. As expected, large proportions of the variance in teacher emotions were located at the class period level, but significant proportions of the variance were also located at the teacher level for each of the emotions, implying that teachers clearly differed from one another in their individual emotional experiences.

**Table 1:** Variance Components at the Teacher and Class Period Level (Study 1).

Emotion	Variance		
	Coefficient	*SE*	Proportion (%)
**Enjoyment**			
Level 2: Teacher (σ^2^_0u_)	.11**	.02	17
Level 1: Class Period (σ^2^_e_)	.53**	.03	83
**Anger**			
Level 2: Teacher (σ^2^_0u_)	.13**	.02	20
Level 1: Class Period (σ^2^_e_)	.53**	.03	80
**Anxiety**			
Level 2: Teacher (σ^2^_0u_)	.10**	.02	26
Level 1: Class Period (σ^2^_e_)	.29**	.03	74

*Note*. *N* = 2,646; 2,668; and 2,649 for enjoyment, anger, and anxiety, respectively, on the class period level, due to missing values. *N* = 135 for all emotions on the teacher level.

** *p* < .01.

Table 2 shows the correlations for the aggregated emotion scores across any two subjects, as well as the averaged correlations across these subject pairs (using Fischer-z-transformation and re-transformation to obtain averages across correlation coefficients, cf. [43]). The average correlations across subjects were of medium size for enjoyment and anger. However, the average correlation between subjects was as high as .68 for anxiety (and as such, significantly higher than the between-subject correlations for both enjoyment and anger, *p*s<.01), suggesting that teacher enjoyment and anger can be considered clearly subject specific, compared to teacher anxiety which depended less on the subject taught.

**Table 2:** Correlations for teaching enjoyment, anger, and anxiety across subjects (Study 1).

Subject Pair	Emotion		
	Enjoyment	Anger	Anxiety
**German—Math**	.52**	.61**	.76**
**German—Science**	.38**	.46**	.62**
**Math—Science**	.34**	.39**	.66**
**Average** ^**a**^	.42**	.49**	.68**

*Note*. *N* = 135.

^a^ To obtain average correlations, we performed a Fisher-z-transformation to the Pearson correlations for the aggregated emotion scores for three subject couples, calculated their arithmetic mean and then performed the inverse Fisher-z-transformation, resulting in values that can again be interpreted like Pearson correlations [cf., 43].

* *p* < .05.

** *p* < .01.

### Discussion

Study 1 was designed to investigate the person and subject specificity of classroom teachers’ enjoyment, anger, and anxiety. In line with previous research on the variation of students’ emotional experiences [25], teachers’ enjoyment, anger, and anxiety fluctuated strongly across diary entries (i.e., class periods). However, it is important to note that variance at this level may also contain measurement error, particularly given that the emotions were assessed using single items. The teachers likely were not able to provide error-free point estimates of their emotional experiences in the previous lesson.

As such, the more pertinent findings of the study are that classroom teachers’ emotions appear to be generally person specific, and that specifically teacher enjoyment and anger also are substantially subject specific. In other words, all three emotions vary from teacher to teacher, but enjoyment and anger also clearly vary within teachers, across the different subjects they teach. These findings are in line with Riggs and Enoch’s (1990) recommendation that teaching efficacy should be assessed in subject specific ways.

In addition, it is worth noting that the degree of subject specificity as found here for teachers seems to be less pronounced than has been reported for students. To reiterate, Goetz et al. [32] virtually found zero correlations between emotional experiences across disparate domains such as German and mathematics, and only correlations of medium size across more closely related subjects such as German and English. Again, one should bear in mind that a direct comparison of Goetz et al.’s and our findings is debatable. Whereas Goetz et al. [32] used multi-item trait-based scales to assess students’ emotions in various subjects, the present study used a repeated-measures diary (state-based) approach to assess teacher emotions in different subjects.

## Study 2: Subject Teachers’ Emotions

The purpose of Study 2 was to replicate and expand upon the results of Study 1. In Study 1, it was not possible to disentangle the teacher from the group of students because teachers instructed only one single group of students per school year. Therefore, in Study 2, we sampled subject teachers. As previously noted, in Germany these teachers typically teach at least two (albeit related) subjects to several different groups of students within one school year. Thus, we sought to additionally analyze the group specificity of teachers’ emotions in the subsequent study. Specifically, we focused on subject teachers who taught mathematics and physics to four different groups of students.

### Method

#### Sample, procedure and measures

The sample consisted of *N =* 85 subject teachers (28.2% female), including 30 secondary school teachers from middle-track schools (Realschule, 53.3% female) and 55 secondary school teachers from upper-track schools (Gymnasium, 14.5% female). On average, teachers were 43.12 years of age (*SD* = 11.23; *range* = 26–61 years), and had an average of 15.35 years of teaching experience (*SD* = 15.36; *range* = 1–40 years). These ages and gender distributions are in line with the documented mathematics and physics teacher population in the state of Bavaria [38].

To assess teachers’ emotions during teaching, the same procedure as in Study 1 was used. Over the course of two weeks (ten school days), teachers filled out the class period diaries after teaching four different groups of students, two in mathematics and two in physics, reporting on an average of 10.2 periods for each emotion.

#### Data structure and strategy of data analysis

As in Study 1, we used hierarchical linear modeling to determine the degree of person specificity of teachers’ emotions. In addition, since this study’s data had in fact a three-level structure (diary entries nested in student groups nested in teachers), the hierarchical approach also allowed for modelling the group-specific within-teacher variability of teacher emotions. Taking the emotion of enjoyment as an example again, the hierarchical linear model is formally represented as follows: The current experience of enjoyment of teacher *k* in group *j* in class period *i* (enjoyment_ijk_), equates to the average experience of enjoyment across the total sample (γ_000_), plus the teacher-specific deviation (ν_0k_), plus the group-specific deviation (u_0jk_), plus the class period specific deviation (e_ijk_). It is assumed that the teacher specific, group specific and class period specific deviations are each normally distributed with a mean of 0, and are independent from each other. As such, the variance of u_0jk_ (σ²_u0_) is an indicator for the intra-individual variance within teachers due to the different groups of students they teach, and the variance of ν_0k_ (σ²_ν0_) is an indicator for the variance between teachers. If σ²_u0_ significantly differs from zero, this indicates that teacher emotions vary systematically within teachers according to the group they are teaching. If σ²_ν0_ significantly differs from zero, this indicates that teacher emotions vary systematically between teachers [40], Chapter 2. We used the software package Mplus (Version 7.11) to process these analyses.

Consistent with study 1 analyses, we applied correlation analyses to determine subject specificity, but also group specificity of classroom teachers’ emotions. To achieve subject-specific emotion scores, we aggregated across all class period entries for mathematics and physics classes that each teacher had reported about. Correspondingly, to achieve group-specific emotion scores, we aggregated across all class period entries for each of the four student groups that the teachers had reported about. We thus obtained correlations across the two subjects as well as across the two mathematics and physics classes that each teacher referred to in the assessment.

### Results

#### Preliminary analyses

The overall mean values across all diary entries for all teachers were *M* = 3.07 for enjoyment (*SD* = 0.48), 1.54 for anger (*SD* = 0.40), and 1.25 for anxiety (*SD* = 0.34). Teachers reported experiencing enjoyment in 96%, anger in 38%, and anxiety in 19% of all their class periods [scoring 2, 3 or 4 on the 4-point agreement scales, see Study 1 [42].

#### Person, subject and group specificity of subject teachers’ emotions

Table 3 presents the variances for enjoyment, anger, and anxiety at the teacher, student group, and class period levels. Again, as expected, large proportions of the variance in teacher emotions were located at the class period level, implying that teachers’ emotions again showed a considerable degree of fluctuation across class periods (however, as noted, the variance at this level also contains measurement error). Clearly, though, teachers again exhibited individual differences in their emotional experiences since significant proportions of the total variance were located at the person level for each emotion. In addition, and most importantly for the present study, significant variance proportions of subject teachers’ emotions were located at the group level. This implies that teachers’ enjoyment, anger, and anxiety systematically varied across the four groups of students they taught.

**Table 3:** Variance components at the teacher, student group, and class period level (Study 2).

Emotion	Variance		
	Coefficient	*SE*	Proportion (%)
**Enjoyment**			
Level 3: Teacher (σ^2^_ν0_)	.19**	.05	28
Level 2: Student Group (σ^2^_u0_)	.07**	.02	10
Level 1: Class Period (σ^2^_e_)	.42**	.03	62
**Anger**			
Level 3: Teacher (σ^2^_ν0_)	.12**	.03	19
Level 2: Student Group (σ^2^_u0_)	.09**	.02	15
Level 1: Class Period (σ^2^_e_)	.41**	.03	66
**Anxiety**			
Level 3: Teacher (σ^2^_ν0_)	.10**	.03	31
Level 2: Student Group (σ^2^_u0_)	.02**	.01	6
Level 1: Class Period (σ^2^_e_)	.20**	.03	63

*Note*. *N* = 1,732; 1,737; and 1,738 for enjoyment, anger, and anxiety, respectively, on the class period level, due to missing values. *N* = 340 for all emotions on the student group level. *N* = 85 for all emotions on the teacher level.

* *p* < .05.

** *p* < .01.

Table 4 shows the correlations for the aggregated emotion scores between mathematics and physics, as well as averaged correlations across the two mathematics and physics student group pairs (using the Fischer-z-transformation and re-transformation for correlation coefficients to obtain averages). These between-subject correlations were small to medium size. Only enjoyment and anxiety significantly differed in their sizes for between-subjects correlations (*ps*<.01), with anxiety showing closer links between subjects than enjoyment. This implies that teachers’ anger and anxiety, but particularly enjoyment, showed some degree of subject specificity even across the closely related subjects of mathematics and physics. Furthermore, in line with results from multi-level analyses, the correlations across student groups fell within a medium range, implying that teacher enjoyment, anger, and anxiety were clearly group specific. These correlations did not differ in size across the three emotions.

**Table 4:** Correlations for teaching enjoyment, anger, and anxiety between mathematics and physics and between student groups (Study 2).

Context	Emotion		
	Enjoyment	Anger	Anxiety
**Subject**	.35**	.51**	.61**
**Student group** ^**a**^	.45**	.43**	.55**

*Note*. *N* = 85.

^a^ To obtain this correlation, we performed a Fisher-z-transformation to the Pearson correlations between the aggregated emotion scores referring to the two mathematics student groups and the two physics student groups each teacher reported about, calculated their arithmetic mean and then performed the inverse Fisher-z-transformation [cf., 43].

** *p* < .01.

### Discussion

Study 2 replicated and extended the results of Study 1. Addressing the teacher/group confound of Study 1, we focused on subject teachers who, in Germany, typically teach at least two (albeit related) subjects to several different groups of students within one school year. Thus, we could additionally analyze the group specificity of teachers’ emotions in this study. In general, the findings on the person specificity of teacher emotions, as demonstrated in Study 1, could be replicated within Study 2. Furthermore, even across the closely related subjects of mathematics and physics, teacher enjoyment again proved to be clearly subject specific, and significantly more so than anxiety. The evidence for subject specificity of anger and anxiety was weaker in this study than in Study 1, which most likely can be attributed to the close proximity of contents within the two subjects of mathematics and physics.

The second key finding from Study 2 is that considerable variability in teachers’ enjoyment, anger, and anxiety, comes from the group of students they teach. This is in line with Raudenbush et al.’s [28] and Ross et al.’s [29] findings on the group specificity of teaching self-efficacy and Kunter et al.’s [7] findings on the context specificity of teaching enthusiasm and enjoyment, as well as with interview data reported by Hargreaves [44] indicating that teachers’ emotional experiences are related to factors resulting from the specific group of students taught.

## General Discussion

Despite the wide-spread assumption that human behavior and experiences are determined both by personality and situational factors [10], researchers to this day continue to debate the actual consistency of human behaviors and experiences across situations [11]. As far as emotions are concerned, it is widely accepted that people differ from one another in the frequency and intensity with which they experience certain emotions, and thus emotions can be considered traits. On the other hand, emotions can also be considered states that strongly fluctuate from moment to moment within each individual [22]. As such, the between-person versus within-person variability remains a particularly intriguing question when it comes to emotions.

The present research utilized the profession of teaching as one particular example of the wide variety of contexts within which these principles of human functioning may be explored. Contextualizing our research in this way had two advantages: It allowed operationalizing our study variables in an applicable manner, and more importantly, the findings bear practical implications for teachers and teaching research (see below). Intriguingly, to date, as far as teacher emotions are concerned, past research seems to have predominantly addressed between-teacher differences, whereas within-teacher variation has rarely been considered.

The present research thus provides a first insight into the question of between and within variability of teacher emotions. To do so, we chose to assess emotions using a diary approach, a technique that acknowledges the fluctuating nature of emotions and holds the promise of providing valid estimates of people’s emotional experiences [37]. Based on existing findings on teaching self-efficacy [28, 29] and student emotions [25, 30–32], we assumed that teachers’ emotions would not only exhibit person-specific variation, but also context-specific variation depending on the academic subject and the specific group of students taught. Sampling both classroom teachers and subject teachers allowed us to test these assumptions.

To begin, it is worth noting that teachers’ emotional experiences in the present studies were overall quite positive. Across both studies, teachers reported experiencing enjoyment in the vast majority of the observed class periods (more than 95% of all class periods). However, teachers also reported experiencing anger in about 40% of their class periods and anxiety in about 20% of all their class periods. This implies that anger and anxiety are also integral parts of teachers’ classroom experiences. These data were similar across our two studies, and intriguingly, also on par with levels of teachers’ enjoyment, anger, and anxiety, as perceived by students [45].

Our first aim was to examine the person specificity of teacher emotions: Are the emotions teachers experience while teaching unique to the individual teacher? The findings across both studies indeed underscored the person specificity of enjoyment, anger, and particularly anxiety.

Our second aim was to examine the context specificity of teachers’ emotions: Do teachers’ emotions vary within teachers according to various contexts they encounter? Our findings are able to offer differentiated insight into this question. Study 1 addressed the subject specificity of emotions for teachers teaching disparate academic subjects (German, mathematics, and the sciences) to a single group of students. Study 2 extended these results by investigating the subject specificity of emotions for teachers teaching related academic subjects (mathematics and physics) with numerous groups of students.

Taken together, our findings provided evidence of the importance of subject for teachers’ enjoyment and anger, but weaker evidence of subject specificity for teachers’ anxiety during teaching. While experiences of anxiety were quite closely linked between subjects in both studies, correlations for enjoyment and anger scores between subjects were maximally as high as .51, which clearly warrants conceptual separation of those subject-specific emotion scores (see also [36, 46], for a similar discussion on the multidimensionality of student self-concept and related motivational variables). Nevertheless, when comparing and contrasting the teacher data presented here with data on the subject specificity of students’ emotions [31, 32], it can be concluded that the academic subject plays a less influential role in teachers’ emotional experiences than it does in students’ emotional experiences.

In addition, Study 2 provided clear evidence for the group specificity of teachers’ emotions, particularly with regard to anger. This finding is consistent with Kunter et al.’s [7] research on the context specificity of teaching enthusiasm and enjoyment, and with Raudenbush et al.’s [28] and Ross et al.’s [29] findings on the group specificity of teaching self-efficacy which most likely is a key appraisal determinant of teachers’ emotions.

Overall, across both of our studies, anxiety tended to show the highest degree of person specificity and the least degree of context specificity. This is in line with the test anxiety tradition that conceptualizes anxiety as being specific to the individual learner, and that typically assessed and treated test anxiety by means that transcend domains [47]. It is also in line with findings from Goetz and colleagues [31] who found that students’ anxiety was more domain general than other student emotions. However, it is worth noting that the findings describing the degree to which anxiety is person specific are mixed. For instance, some studies suggest that students’ anxiety is no more person specific than enjoyment [25, 32].

Taken as a whole, the results of this research confirm the notion that teacher emotions, like other human experiences and behaviors, are a function of personality and situational factors [48]. Across both studies, our findings suggest that trait dispositions seem to play an important role for teacher emotions, but there is also considerable within-teacher variance due to contextual factors (subject and group of students taught).

### Limitations and Directions for Future Research

In this research, data was collected from both primary school (classroom) and secondary school (subject) teachers. The consistency of the findings across these two independent studies supports their external validity and lends credibility to our conclusions.

Nevertheless, several limitations to the present research also warrant attention because of their implications for future studies and educational practice. First, the current research used self-report data to assess emotions. Self-reports are highly useful sources of information with regard to how people perceive and interpret situations [49, 50], but their validity has been questioned by some [51], and the use of self-reports always carries with it the risk that people will answer in a socially desirable manner [49].

Second, it is worth noting that our class period diary approach to measuring emotions is an uncommon assessment strategy. By asking about the emotions experienced during teaching directly after a class period, we made a compromise between momentary assessment (e.g., experience sampling) and recall-based assessment of emotions [37, 52]. We chose this approach because it is less intrusive than momentary assessment and accounts for the naturally occurring fluctuations in emotions. We believe it closely reflects the true levels of emotions experienced during teaching. Nevertheless, it remains open to question whether diary measures of emotions can adequately overcome the problems involved with classical recall-based measures. Recall-based measures may be influenced by the most recent emotional experience or particularly salient experiences, and they may be biased by memory limitations, global heuristics, implicit theories of emotions, and beliefs about one’s emotionality [53, 54]. Future research on teacher emotions should therefore complement the diary approach by using alternative measures, such as physiological indicators or behavioral observation [55, 56].

Lastly, while the present research examined the sources of variance in teacher emotions in terms of individual and context-related differences, it did not investigate the mechanisms that could be responsible for these differences. For example, the variation in teacher emotions due to individual differences may be due to differences in teachers’ individual temperament and general trait emotions such as positive or negative emotionality. Moreover, differences attributable to academic subject may be due to differences in teachers’ appraisals of these subjects, whereas differences attributable to the group of students taught may be due to characteristics of the group, such as students’ discipline, engagement, or academic performance [3]. As such, future research should examine the mechanisms responsible for variation in teacher emotions at the teacher, subject, and group level.

### Implications for Practice

Our findings have important implications for the assessment of teacher emotions and for the design of interventions aimed at improving teachers’ emotional lives. Our studies suggest that teacher emotions are, to a large degree, person specific. As such, appropriate assessments of teacher emotions may be those that generalize across contexts and target the individual themselves (e.g., “How much enjoyment, anger, and anxiety do you typically feel when teaching”). Similarly, this person specificity underscores the promise of individually-tailored intervention programs to help teachers develop individual strategies to regulate their emotions. For example, teachers could—with training—learn to use problem- and emotion-focused strategies to up-regulate their enjoyment and down-regulate their anger and anxiety during teaching most generally (see e.g., [57] on teachers' sponaneous use of emotion regulation strategies in the classroom, or [58]).

Beyond such general approaches, our findings also imply that both assessment and intervention could profit from considering subject specific and group specific factors. As such, assessments targeting subject- and/or group-specific teacher emotions (e.g., “How much enjoyment/anger/anxiety do you feel when teaching subject x/this particular group of students”) might be even more meaningful than context-general assessments. Interestingly, the degree to which student motivation can and should be understood as context specific versus generalized across contexts is a similarly debated topic [59, 60]. To illustrate, Marsh and Yeung [61] argued that ‘‘researchers should consider multiple dimensions of self-concept particularly relevant to the concerns of their research, supplemented, perhaps, by more general measures” (p. 526). The same may apply for the assessment of teacher emotions.

With respect to intervention, the findings imply that teachers should be instructed to focus on what causes their emotions when teaching a particular subject and/or a particular group of students. For example, this may include classroom activities specifically tailored to teach certain challenging content areas, or classroom management strategies that are specifically designed to tackle certain problems arising within a particular group of students.

To conclude, we encourage investigators to consider both person- and context-specific variables when investigating teachers’ emotions and when designing assessments and intervention programs. This should be conducive to the validity of measures of teacher emotions and to the quality of interventions targeting these emotions.
